# HSP70 interacting protein prevents the accumulation of inclusions in polyglutamine disease^1^

**DOI:** 10.1111/j.1471-4159.2008.05847.x

**Published:** 2009-02

**Authors:** Joanna L Howarth, Colin P J Glover, James B Uney

**Affiliations:** Henry Wellcome Laboratories for Integrated Neuroscience and Endocrinology, Dorothy Hodgkin Building, University of BristolBristol, UK

**Keywords:** adenoviral, heat shock protein 70, HSC70, HSP70 interacting protein, Huntington’s disease, polyglutamine

## Abstract

Heat shock proteins (HSPs) are associated with the proteinaceous inclusions that characterise many neurodegenerative diseases. This suggests they may be associated with disease aetiology and/or represents an attempt to remove abnormal protein aggregates. In this study the adenoviral mediated over-expression of HSP70 interacting protein (HIP) alone was shown to significantly reduce inclusion formation in both an *in vitro* model of Spinal Bulbar Muscular Atrophy and a primary neuronal model of polyglutamine disease. Experiments to determine the mechanism of action showed that: denatured luciferase activity (a measure of protein refolding) was not increased in the presence of HIP alone but was increased when HIP was co-expressed with HSP70 or Heat Shock cognate protein 70 (HSC70); the expression of polyglutamine inclusions in cortical neurons mediated an increase in the levels of HSC70 but not HSP70. Our data suggest that HIP may prevent inclusion formation by facilitating the constitutive HSC70 refolding cycle and possibly by preventing aggregation. HIP expression is not increased following stress and its over-expression may therefore reduce toxic polyglutamine aggregation events and contribute to an effective therapeutic strategy.

Polyglutamine (PolyQ) disorders such as Spinal and Bulbar Muscular Atrophy (SBMA) and Huntington’s disease are caused by an expansion in the glutamine tri-nucleotide (CAG) repeat region of affected genes (Fu *et al.* 1991; La Spada and Taylor 2003). The expansion results in the production of mutant proteins which aggregate and form insoluble inclusions within affected neurons (Ordway *et al.* 1997). The mechanism by which the mutant proteins mediate neuronal cell death remains uncertain. Insoluble nuclear or cytoplasmic disease protein inclusions were initially thought to activate apoptotic pathways and/or alter patterns of gene transcription (Lipinski and Yuan 2004). However, studies have suggested that small monomers of mutant protein are responsible for the observed neuronal toxicity and that insoluble inclusions are formed because of the sequestration of these monomeric mutant protein (Watase *et al.* 2002; Yu *et al.* 2002; Arrasate *et al.* 2004).

Molecular chaperones play a key role in protein synthesis and biogenesis (Bukau *et al.* 2006) and they are found bound to the insoluble protein inclusions that characterise neurodegenerative diseases, including Alzheimer’s Disease, Parkinson’s Disease and many PolyQ diseases (Cummings *et al.* 1998; Bailey *et al.* 2002; Petrucelli *et al.* 2004). Often the characteristic intracellular inclusions of each disease are associated with the small heat shock protein ubiquitin and heat shock protein 70 (HSP70) (Muchowski and Wacker 2005). HSPs have therefore been hypothesised to be associated with the aetiology of these diseases and perhaps more likely that their up-regulation represents an attempt to refold or remove the abnormal protein aggregates (Chai *et al.* 1999). Consistent with the latter hypothesis are the observations that over-expression of HSP70 and proteins that facilitate targeting to the ubiquitin-proteasome system (UPS) suppress aggregate formation in models of Huntington’s disease (Howarth *et al.* 2007). The role small molecular co-chaperones play in facilitating the removal of protein inclusions has also recently been studied (Jana and Nukina 2005; Jana *et al.* 2005). HSP70 interacting protein (HIP/p48) represents a unique class of co-chaperones that bind the ATPase domain of HSC70 and HSP70, stabilizing the complex formed with ADP and thus facilitates refolding of substrate proteins (Hohfeld *et al.* 1995; Bruce and Churchich 1997). In addition, HIP is reported to be a chaperone in its own right, binding to unfolded proteins and preventing their aggregation but not mediating refolding (Bruce and Churchich 1997). Structural analysis of HIP demonstrated that this co-chaperone combines structural elements found in HSC70 and other HSC70/HSP90 associated co-chaperones, including tetracopeptide repeat regions (Irmer and Hohfeld 1997) and several repeats of the tetrapeptide GGMP (Hohfeld *et al.* 1995). Deletion studies revealed that the HSP70-binding domain and the homo-oligomerization domain of HIP are required for HSP70-mediated reactivation of denatured firefly luciferase. The evolutionary conservation of such domains suggests that HIP plays an important role in HSC70/HSP90 regulation, enabling the formation of multimeric chaperone complexes with their substrates (Irmer and Hohfeld 1997; Bedard *et al.* 2007). However the role of HIP within a disease context has not yet been investigated. We have used powerful adenoviral (Ad) gene delivery systems and *in vitro* models of SBMA and PolyQ disease to study HIP function and assess its ability to suppress the formation of insoluble protein inclusions.

## Materials and methods

### Transfection of androgen receptor

Mouse neuroblastoma (N2a) cells were grown in Dulbecco’s Minimum Essential Medium with 5000 mg/L Glucose (DMEM; Sigma, St Louis, MO, USA), supplemented with 10% (v/v) heat inactivated fetal calf serum (FCS; Gibco, Rockville, MD, USA), 100 U/mL penicillin, 0.1 mg/mL streptomycin and 2 mM l-glutamine (both Sigma) in a humidified, 5% CO_2_ atmosphere, at 37°C. Cells were transfected (using Lipofectamine) with vectors encoding the human Androgen Receptor construct plus 20 CAG repeats (hAR) or an expanded ‘knock-in’ construct (hARk) containing 51 repeats. Cells were subsequently transduced with adenoviral vectors expressing HSP70, HSC70, HSP40 or HIP. After 24 h cells were incubated in Phenol Red Free/Dextran Coated Charcoal (PFR/DCC) media for a further 24 h, prior to stimulation with 50 nM testosterone.

### Primary cortical neuron cultures

Primary cortical tissue dissected from Wistar rat embryos at embryonic day E18 were cultured following a protocol described previously (Howarth *et al.* 2007). Cells were plated at a density of 100 000 cells per well of a standard 24 or 4 well plate (Nunc, Naperville, IL, USA) and cultures were transduced with adenoviral vectors after 5 days.

### Virus production

E1 deleted adenoviral (Ad) vectors expressing various heat shock proteins and co-chaperones were produced by homologous recombination in HEK293 cells according to standard techniques (Harding *et al.* 1998). Additional Ad vectors expressing CAG repeat fragments tagged with green fluorescent protein (GFP) (QnGFP) from the neuron-specific synapsin promoter were used to produce an *in vitro* model of PolyQ disease in primary cortical neuron as previously described (Howarth *et al.* 2007). Ad vectors expressing an expanded Exon1 construct of the Huntingtin gene containing 103 CAG repeats (HttEx1-Q103EGFP), a kind gift from Dr Wyttenbach (Firdaus *et al.* 2006), were amplified by the same method.

### Immunofluoresence microscopy

Following fixation in methanol, transfected cells were incubated with anti-androgen receptor antibody, AR N20 (1 : 150, Santa Cruz Biotechnology, Santa Cruz, CA, USA). FITC-conjugated anti-rabbit antibody (1 : 200, Jackson Immuno-Research, West Grove, PA, USA) enabled visualisation of SBMA inclusions within cells. Cells were mounted with non-quenching medium (Vectashield, Vector Laboratories, Peterborough, UK) and images captured using an Inverted Leica Confocal Imaging system. All counts of cell inclusions were performed blind and recorded the presence or absence of any inclusions (regardless of size or number) in a cell. Cortical neurons were fixed in 4% paraformaldehyde, before incubation with anti-HSC70 antibody (1 : 200, SPA-815 Stressgen, Collegeville, PA, USA).

### Luciferase refolding assay

Neuroblastoma cells were co-transduced with Ad vectors expressing luciferase and various heat shock proteins and/or co-chaperones. After 3 days, cells were subjected to heat shock at 46°C and left to recover for 0–60 min in a 37°C incubator and luciferase assays then carried out (Promega, Madison, WI, USA). Luciferase activity was also measured in control (undenatured) cells. Similar experiments were performed in primary cortical neurons using a milder temperature of 42°C which yielded the most reproducible results.

### Filter-trap assays

Cells were lysed with sodium dodecyl sulfate (SDS) lysis buffer and centrifuged to obtain insoluble and soluble fractions as previously described (Bailey *et al.* 2002). Samples were applied onto nitrocellulose or cellulose acetate membranes under vacuum using slot-blot apparatus. Proteins within each fraction were detected by probing with antibodies against GFP (Roche Molecular Biochemicals, Indianapolis, IN, USA), Ubiquitin (Ubi1, Abcam Inc., Cambridge, MA, USA) or AR (N20, Santa Cruz) and visualised using *Enhanced* Chemiluminescence reagent.

### Western blots

Neuroblastoma cells were transduced with Ad vectors [at a multiplicity of infection (MOI) of 100 viral particles per cell] and lysed after 48 h using Radio Immuno Precipitation Assay Buffer [1× phosphate-buffered saline, 1% Nonidet P-40 (Roche Applied Science, Mannheim, Germany), 0.5% sodium deoxycholate, 0.1% SDS (all Sigma)] containing protease inhibitors phenyl-methyl-sulphonyl-fluoride (PMSF; 100 μg/mL), aprotinin (70 μg/mL) and sodium orthovanadate (1 mM). Samples from primary cortical neuron cultures were lysed 8 days post-transduction with Ad vectors. All samples were resolved by SDS gel electrophoresis at the appropriate percentage acrylamide. Proteins were transferred onto polyvinylidene difluoride) membrane (Roche), probed with primary antibodies against HSC70 (SPA-815, Stressgen, 1 : 10 000), HSP70 (SPA-810, Stressgen, 1 : 40 000), HSP40 (SPA-450, Stressgen 1 : 10 000), HIP (R-19, Santa Cruz, 1 : 1000), or Alpha Tubulin (Sigma, 1 : 1000) and visualised using ECL detection via horseradish peroxidase conjugated secondary antibodies. Co-immuno precipitation assay methods are detailed in Appendix S1.

## Results

### Estimating HSP mediated protein refolding

**Fig. 1:**
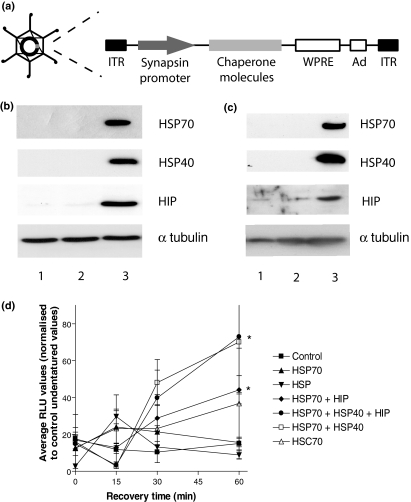
Adenoviral mediated expression of co-chaperones*.* (a) Schematic diagram of adenoviral vectors used in this study: the neuron specific synapsin promoter was used to drive expression; WPRE, woodchuck hepatitis virus post-transcriptional regulatory element. Western blotting verified the Ad mediated expression of HIP, HSP70 and HSP40 (Lane 3) in (b) N2a cells and (c) in primary cortical neurons. Lanes 1 and 2 denote control (untransduced) cells and cells transduced with an adenoviral control ‘empty’ vector (AdØ), respectively. Alpha tubulin was used as a loading control. (d) N2a cells were co-transduced with Ad vectors expressing luciferase and HSPs and luciferase activity was measured following heat shock: RLU, Relative light units. Statistical analysis was carried out by ANOVA followed by post-hoc *t*-tests, *p < 0.05.

Adenoviral vectors expressing HIP, HSP70 and HSP40 (all human) were used to transduce cells (Fig. 1a). Western blots confirmed high protein expression in both N2a cells and primary cortical neurons following viral transduction compared to the low endogenous expression of each HSP/co-chaperone (Fig. 1b and c). In addition the co-expression of multiple chaperones mediated similar levels of expression (data not shown). To estimate refolding activity N2a cells (a cell line used extensively in similar studies) were transduced with Ad-vectors expressing chaperones and luciferase and then heat shocked. When expressed alone, HIP did not significantly increase luciferase activity after the initial denaturing stress (Fig. 1d). However, a statistically significant increase in luciferase activity (*p* < 0.05) was observed when cells were co-transduced with HSP70 and HIP. Similar results were also obtained following co-transduction with HSP70 and HSP40 in the presence and absence of HIP.

### Assessing the effect of HIP on PolyQ inclusion formation

**Fig. 2:**
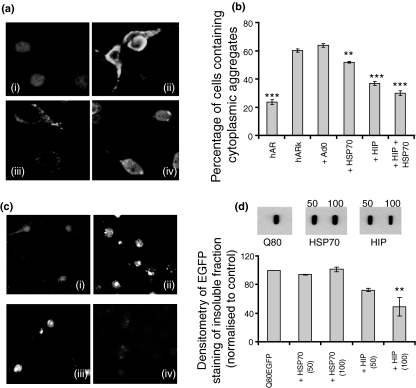
Reduction of aggregates in models of PolyQ disease. (a) Photomicrographs of mouse neuroblastoma (N2a) cells transfected with control hAR plasmid (i), the expanded hARk SBMA construct (ii), hARk transfection followed by transduction with Ad vectors expressing HSP70 (iii) or HIP (iv). (b) Following each treatment, the number of cells with and without aggregates was counted (blind). The results are presented as the percentage of cells containing aggregates relative to the untreated control ± SEM (*n* = 12). Chaperone transduced cells were compared to the hARk control and statistical analysis was carried out by anova followed by *post-hoc t*-tests. (c) Cortical neurons were transduced with Ad vectors expressing Q19GFP (i), Q80GFP (ii), Q80GFP + HSP70 (iii) or Q80GFP + HIP (iv). (d) Lysates from cortical neurons transduced with HSP70 or HIP at a viral MOI of 50 or 100 were fractionated and applied to cellulose acetate membranes and probed with an anti-GFP primary antibody. Membranes were analysed by densitometry to quantify changes in insoluble inclusions. Statistical analysis was carried out by anova followed by *post-hoc t*-tests, **p < 0.01, ***p < 0.001.

Cells were transfected with SBMA plasmids, hAR and hARk (containing 20 and 51 polyQ repeats respectively) and immunocytochemical analysis was used to estimate inclusion formation (Fig. 2a). This SBMA model of polyQ disease was used to perform preliminary experiments to assess the effectiveness of co-chaperones at reducing the number of cells containing polyQ inclusions. Our results demonstrated that transfection of HIP resulted in a significant suppression of inclusion formation, (*p* < 0.001) and that this was greater than that mediated by HSP70 alone (*p* < 0.01) (Fig. 2b). Transduction with Ad vectors expressing HIP in conjunction with HSP70 also mediated a significant reduction in the number of cells containing cytosolic inclusions (*p* < 0.001). During these studies cell death was also assessed by 4′-6-Diamidino-2-phenylindole (DAPI) staining and cell counts and the expression of SBMA plasmids alone or together with HIP did not lead to a significant increase in the number of pyknotic nuclei or alter cell numbers.

Inclusion formation was also estimated in a more complex primary neuronal model of PolyQ disease to better model events in the CNS. Cortical neurons were transduced with Ad vectors expressing Q19EGFP and Q80EGFP fusion proteins under the control of a neuron specific promoter. Following HIP expression, fluorescence microscopy revealed a reduction in the size and number of aggregates in cortical neurons (Fig. 2c). Filter-trap assays were performed to quantify the level of insoluble protein following the transduction of cells with HSP70 or HIP (Fig. 2d). Densitometry results showed that transduction with HIP alone at the higher viral multiplicity of infection (of 100 particles per cell) led to a highly significant decrease (*p* < 0.001) in the formation of insoluble Q80EGFP, with a concomitant increase in the soluble fraction (Fig. 2d). In addition the level of cell death was not altered following expression of Q80EGFP and/or HIP.

### HIP expression does not increase ubiquitin-mediated removal of PolyQ proteins

**Fig. 3:**
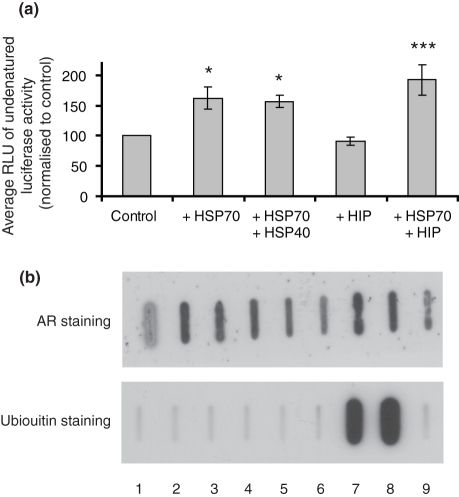
HIP does not increase the ubiquitination of insoluble PolyQ aggregates*.* (a) Undenatured luciferase activity was measure in N2a cells following Ad-mediated expression of HSP70, HSP40 and HIP. Statistical analysis was performed by anova followed by *post-hoc t*-tests. ***p < 0.001, *p < 0.05. (b) Slot-blot assays were performed on N2a cells expressing SBMA plasmids and various chaperone molecules. The levels of androgen receptor and ubiquitin in the insoluble protein fraction following expression of hAR (1) and hARk alone (2) and in the presence of AdØ (3), HSP70 (4), HSC70 (5), HSP40 (6), HSJ1a (7), HSJ1b (8) and HIP (9), were determined using anti-AR or anti-ubiquitin antibodies.

Analysis of steady-state luciferase levels in non-heat shocked control cells showed that HIP does not alter steady-state luciferase levels (Fig. 3a). Conversely HSP70, either alone or in conjunction with HIP, led to a significant increase in steady state luciferase activity (*p* < 0.001). These results were supported by slot-blot experiments performed to investigate ubiquitin-mediated removal of the hARk construct via the ubiquitin proteosome system. These results showed that transfection of hARk produced high levels of insoluble AR compared with hAR, but that these proteins were not ubiquitinated. Similarly the presence of HIP did not increase the ubiquitination of proteins present in the insoluble fraction, whereas HSJ1a and HSJ1b (HSP40 family members that contain ubiquitin interacting motifs known for their involvement in proteasomal degradation of misfolded proteins) did increase the presence of ubiquitinated proteins in the insoluble fraction (Fig. 3b). Similar results were obtained following Ad-mediated expression of HIP in cells expressing Q80EGFP. Taken together these observations suggest there is no degradation/removal of steady-state luciferase levels following the expression of HIP.

### Expanded polyglutamine repeats increase HSC70 expression

**Fig. 4:**
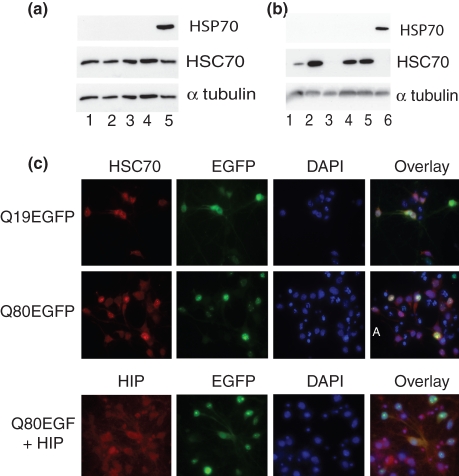
Expression of expanded PolyQ repeats increase HSC70 expression. (a) Western blot analysis was performed on N2a cells transfected with hAR (2), hARk (3), and cells transduced with Ad vectors expressing HSC70 (4), or HSP70 (5). Polyvinylidene difluoride membranes were probed with antibodies against HSP70, HSC70 and alpha tubulin and compared to levels in untransfected control cells (1). (b) Western blot analysis performed on cortical neurons transduced with Ad vectors expressing Q19GFP (1), Q80GFP (2), EGFP (3), HttEx1-Q103EGFP (4), HSC70 (5) and HSP70 (6). (c) Photomicrographs of cortical neurons transduced with Ad vectors expressing Q19GFP or Q80GFP were stained with HSC70 antibody and DAPI to show nuclear staining. Additional cultures co-transduced with Q80GFP and HIP were stained with an anti-HIP antibody. Overlay images indicate co-localisation of HSC70 with polyQ aggregates and HIP with diffuse EGFP monomers.

The reduction in PolyQ inclusion formation may indicate that HIP facilitates HSP70-mediated refolding. To assess this possibility the endogenous levels of HSP70 family members in N2a cells and primary cortical neurons were measured. Western blotting showed that of HSC70 was expressed at high constitutive levels in N2a cells and that expression of hARk did not increase these levels further or induce the expression of HSP70 (Fig. 4a). In cortical neurons endogenous levels of HSC70 could also be detected, however, Ad-mediated expression of Q80EGFP or HttQ103EGFP resulted in an increase in HSC70 but not induction of HSP70 (Fig. 4b). Similar results were observed following immunocytochemical studies on cortical neurons expressing Q19EGFP or Q80EFGP where the expression of Q80EGFP led to an increase of HSC70 which co-localised with PolyQ aggregates (Fig. 4c). In contrast viral-mediated over-expression of HIP in cortical neurons reduced the number of PolyQ aggregates. Inclusion formation appeared to be inversely correlated with the level of HIP expression such that co-localisation was only detected in cells showing diffuse enhanced green fluorescent protein (EGFP) staining (Fig. 4c). Similar results were also observed by co-immunoprecipitation studies which showed that HIP associated with Q80EGFP and redued the amount of insoluble Q80EGFP formed (Fig. S1).

**Fig. 5:**
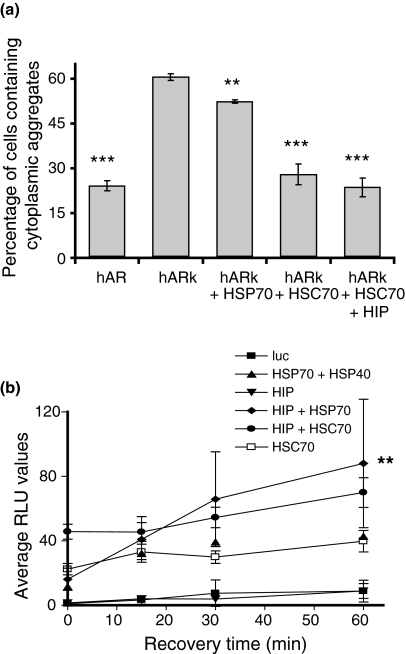
Expression of expanded PolyQ repeats increase HSC70 expression. (a) N2a cells were transfected with hAR or hARk constructs alone or with hARk in conjunction with Ad vectors expressing HSP70, HSC70 and/or HIP. The percentage of cells containing aggregates relative to the untreated control was assessed. (b) Primary cortical neurons were co-transduced with Ad vectors expressing luciferase and HSP70, HSC70, HSP40 and/or HIP. Neurons were then subjected to heat shock and allowed to recover before analysis of denatured luciferase activity. Statistical analysis was carried out by anova followed by *post-hoc t*-tests, **p < 0.01, ***p < 0.001.

Additional studies in our SBMA model also confirmed that the Ad-mediated expression of HSC70 reduces the percentage of cells containing cytoplasmic PolyQ inclusions (Fig. 5a). Denatured luciferase refolding assays were then performed in primary cortical neurons (Fig. 5b) and the results showed that HIP when expressed alone did not mediate an increase in luciferase activity. However a significant increase in denatured luciferase activity (*p* < 0.01) was observed following the Ad mediated expression of HSP70 and in the presence of HSC70, HIP also mediated a small, but non-significant increase in refolding.

## Discussion

Polyglutamine diseases are characterised by insoluble protein inclusions that are thought to eventually contribute to the death of neurons. In recent years strategies which may increase the removal of disease proteins (e.g. the stimulation of protein refolding and activation of proteasomal degradation pathways) have been examined. In this study we investigated for the first time the effect of expressing HIP, a positive regulator of HSP70 and HSC70, on the formation of inclusions containing PolyQ expansions. The results showed that viral-mediated over-expression of HIP alone significantly reduced inclusion formation in two polyglutamine disease models. Investigations into the mechanism of action showed that HIP could only facilitate luciferase refolding in the presence of HSP70 or HSC70. Interestingly, the expression of an expanded PolyQ tract in primary neurons increased the expression of HSC70 but not HSP70. Experiments using the SBMA model showed that the adenoviral mediated expression of HSC70 was more effective than HSP70 at reducing inclusion formation and that HIP facilitated HSC70/HSP70 mediated refolding activity. There was no evidence that the expression of HIP increased the ubiquitination of PolyQ substrates. Our data therefore suggest that HIP prevents inclusion formation by facilitating constitutive HSC70 chaperone activity and not by targeting to the ubiquitin proteosome system.

Prior work has shown that HIP prevented the aggregation of unfolded polypeptides but did not mediate refolding of denatured proteins (Hohfeld *et al.* 1995; Bruce and Churchich 1997). It was further hypothesised that HIP may contribute to the stabilisation of unfolded polypeptide substrates prior to interacting with HSC/HSP70 (ibid). HIP was originally reported to positively regulate HSC70 by binding and stabilising the ATPase domain when in the ADP-bound state. By performing this function, HIP is currently considered to antagonize the substrate discharging function of another co-chaperone molecule, BAG-1 (Nollen *et al.* 2001). Thus HIP expression prevents the BAG-1 mediated slowing of the ATPase cycle of HSC70 (Hohfeld and Jentsch 1997) and ensures optimal chaperoning of misfolded proteins. We found the expression of the co-chaperone molecules HSP40 and HIP led to a reduction in insoluble PolyQ proteins by enhancing the refolding of misfolded proteins. In the primary cortical neuron model, the expression of polyglutamine proteins resulted in a significant increase in HSC70 but this alone was not sufficient to reduce inclusions formation. This is likely to be because of the relatively weak ATPase activity of HSP70 family members. The introduction of co-chaperones enhances the efficiency of the HSC70 refolding cycle and HSP40 has been shown previously to decrease inclusion formation via this mechanism. The results of this study propose a similar role for HIP, which acts as a nucleotide exchange factor, enhancing substrate processing by HSC70 family members (Hohfeld *et al.* 1995; Bruce and Churchich 1997).

In summary our data suggest that HIP suppresses the formation of insoluble PolyQ inclusions by: (i) facilitating the HSC70 refolding cycle, acting in concert with enhanced levels of endogenous HSC70 following PolyQ expression; (ii) binding to unfolded polyglutamine proteins and preventing their aggregation. Considering HSC70 levels may be elevated in the neurons expressing disease aggregates, increasing HIP/co-chaperone expression could prove to be a useful therapeutic strategy for the treatment of PolyQ diseases.

## Abbreviations used

hAR: human androgen receptor
HIP: HSP70 interacting protein
HSC70: Heat Shock cognate protein 70
HSP: heat shock protein
N2a: neuroblastoma
PolyQ: polyglutamine
SBMA: spinal and bulbar muscular atrophy
SDS: sodium dodecyl sulfate

## Supporting information

**Figure S1.** EGFP co-immunoprecipitation assay.

**Appendix S1.** Co-immuno precipitation assays.
