# Supplementary material for: HSP70 interacting protein prevents the accumulation of inclusions in polyglutamine disease1
Source: J Neurochem. 2009 Feb;108(4):945–51. doi: 10.1111/j.1471-4159.2008.05847.x (PMC2779461; doi:10.1111/j.1471-4159.2008.05847.x)
Supplement: Supplementary file 2 [file jnc_5847_SD2.doc]

***Supplemental Figure 1: EGFP co-immunoprecipitation assay***

EGFP co-immunoprecipitation assays were performed on cortical neurons transduced with Ad vectors expressing Q80EGFP (alone or together with Hip). The presence of high molecular weight inclusions following Q80EGFP expression is reduced by viral-mediated Hip expression and a lower molecular weight band is detected. The binding of Hip to EGFP is also demonstrated. Untransduced neurons were used as a control.

***Supplemental methods: Co-Immuno precipitation assays***

Co-immunoprecipitation was performed using a ProFound co-immunoprecipitation kit (Pierce, Perbio Science, UK). In brief, cortical neurons transduced with Ad vectors expressing Q80EGFP (alone or together with Hip) were lysed 8 days after transduction with co-immunoprecipitation lysis buffer (50 mmol/l 4-(2-hydroxyethyl)-1-piperazineethanesulfonic acid pH 7.9, 250 mmol/l NaCl, 1 mmol/l

EDTA, 1 mmol/l Na3VO4, 20 mmol/l *β*-glycerophosphate, 5 mmol/l 1,4-dithiothreitol, 0.5% Igepal) containing protease inhibitors (Protease cocktail; Roche). Cell lysates were incubated with Aminolink plus coupling gel coupled to GFP antibody (Roche) at 4ºC. Samples were boiled in sodium dodecyl sulfate polyacrylamide gel electrophoresis loading buffer to elute associated proteins and separated by 10% acrylamide gels. Proteins were transferred onto polyvinylidene fluoride membranes, which were then probed with anti-GFP (1:1,000, Roche) and anti-Hip (1:1,000; Santa Cruz) antibodies. Untransduced neurons were used as a control.
